# The Origin of Individual Differences in the Course and Severity of Diseases

**DOI:** 10.1100/tsw.2006.278

**Published:** 2006-12-28

**Authors:** Sergey N. Rumyantsev

**Affiliations:** Andent, Inc., Waukegan, IL, USA

**Keywords:** biodiversity, clinical immunology, heterozygosity, individuality, severity of diseases

## Abstract

Any disease manifests itself individually either by a bright or restricted spectrum of clinical signs and resultant clinical courses that range from asymptomatic to deadly, from acute to chronic, etc. Until recently, the origin of this kind of biodiversity was poorly investigated and understood, but advances in immunology — especially in identifying constitutional (genetic) mechanisms of immunity — have contributed to our understanding of the origin of individual diversity in diseases. Any disease destroys only focal areas in the affected organisms, and the amounts, sizes, and distribution of such focal lesions vary from patient to patient. In a population predilected to a relevant pathogenic agent, individuals can be conveniently divided into three categories: totally resistant organisms that contain no susceptible structures and are not affected; mildly susceptible organisms in which a few foci appear and in which the disease runs a benign course; organisms in which the number of constitutionally susceptible structures is high and the disease develops in a severe form. The diversity is formed by the mating of genetically different parents that determines the differences in susceptibility of genetically various parts of the descendant organism.

## VARIABILITY IN INFECTIOUS DISEASES

Any disease can manifest itself by either a bright or restricted spectrum of individual expression of its specific clinical signs and resultant clinical courses that range from asymptomatic to deadly, from acute to chronic, etc. Until recently[1], the origin of this kind of human biodiversity has not been investigated and understood.

### Observations

Every infectious disease affects only some areas of the infected organism, forming focal lesions of individually variable distribution[2,3]. The focal lesion of cutis is seen, for instance, during poxvirus infection. It consists of a number of skin lesions scattered over the surface of the body, each of which is the seat of inflammation so intense as to lead to the formation of small abscesses in the course of 4–5 days (Fig. 1). Beyond the edge of the lesions, the skin is normal. The number of lesions present may be less than a dozen in a minor case of illness, or they may number in the thousands. The smallpox rash is most severe on the face, then the hands and upper extremity. From the hands upwards, the scabs diminished in density. On the front part of the trunk they are scarce, especially on the abdomen. The areas adjacent to the specific damage are found almost intact, though all the skin is presumably homogeneous[3,4,5]. In a severe course of disease, lesions may be set so closely as to conceal almost the whole cutaneous surface[5], forming the heaviest kind of the infection known as the "black smallpox".

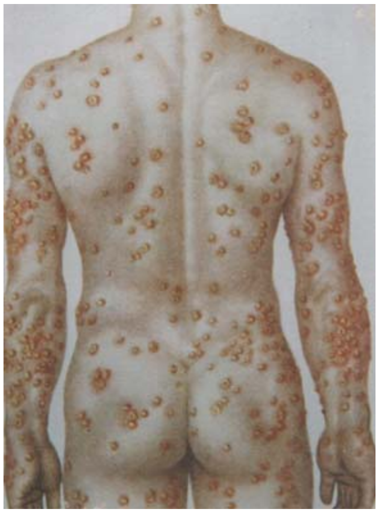

**FIGURE 1**. Mosaic focal distributions of specific lesions in a case of human smallpox.

Poliovirus infection targets nerve cells at random, although the virus is present throughout the nervous system[6]. The brain and spinal cord are involved in every case of poliomyelitis, but not every area of these organs is susceptible to the virus. This can only mean that nerve cells in certain areas are susceptible to viral damage, whereas those in other areas are not. In the worst cases, nearly every skeletal muscle may be paralyzed. In the mildest cases, paralysis may be limited to part of one muscle. All degrees and combinations of paralyses can be observed[4].

Mosaic distribution of damages is also characteristic of hepatitis, tuberculosis, and many other infections that chiefly affect internal organs[4,7]. The major recognized forms of Q fever are pneumonia, hepatitis, meningoencephalitis, vascular infection, bone infection, osteomyelitis, endocarditis, unexplained fever, and flu-like form[8]. Typhoid and paratyphoid fever produce inflammatory foci in many organs and, in some areas, necrosis and sloughing of tissues. Necrotic or inflammatory foci are commonly seen in the spleen and the liver, and sometimes in the kidney, the meninges, the bones, the endocardium, the joints, and many other portions of the body[4].

In bacillary dysentery, the specific pathological changes are located in the colonic mucosa and consist of random patches of grayish membrane composed of thickened, necrotic mucosa. In severe cases, the disease may produce the destruction of the entire large bowel. In less-severe cases, the areas of necrosis of the mucous membrane are less extensive, with small, scattered ulcers or nodular crusted lesions appearing on the bowel wall[4]. Focal bone lesions were characteristic of some infections in bears during the Pleistocene era, circa 10,000 years B.C.[9].

According to Black-Schaffer et al.[10], the number and size of infectious lesions depend on two factors: the patient reactivity and the potency of the infecting strain. This commonly used statement does not explain why these factors do not work in all parts of a body. The examples considered above illustrate the manifestation of the mosaic configuration within affected homogenous structures. Evidently, the ubiquitous phagocytes and immunoglobulins are not functional in this phenomenon. The focal nature of infection is not explained by the lack of uniform predilection of micro-organisms for the organism. Every infection in its pre-eruptive stage is a septicemia and the causative agents are circulated everywhere in the affected tissue, but they induce specific eruption only in susceptible areas of the tissue. In such cases, the infectious agent is found within both the typical damaged elements and the adjacent areas that have been left intact[3,4,11]. Recent advances in immunology — especially in identifying the constitutional (genetic) agents of immunity and its origin — have contributed to our understanding of the origin of individual diversity in diseases. According to previous studies[1,2], the mechanisms involved in the discussed phenomenon are of constitutional origin.

## Constitutional Immunity

### *The Kinds of Immunity*

Immunity is the state of being immune; that is, having a degree of natural or acquired resistance to a disease[12]. Immunity involves an extremely complex set of surveillance systems that provide a remarkable group of agents, each geared to the maintenance of a healthy existence. Some of the agents and systems are already well known.

Vertebrate animals possess several systems of immunogenesis (Fig. 2). Constitutional and phagocytic mechanisms serve as a first line of defense. If they are ineffective, the attacked organism can be rescued by the influence of specific immunoglobulins and other inducible agents that are produced mainly by the lymphatic system of reactive immunity.

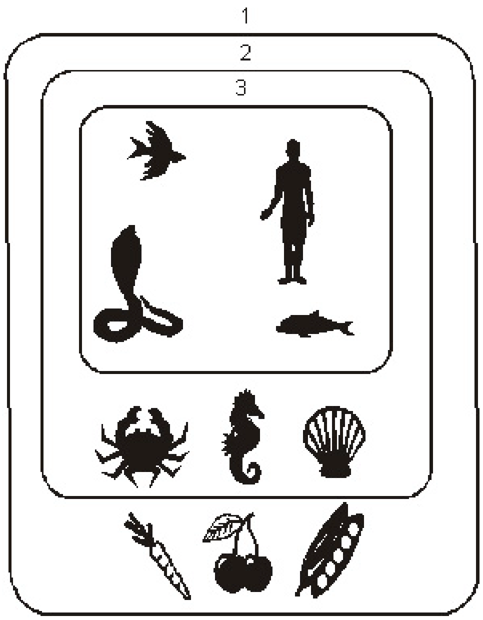

**FIGURE 2.** The main systems of immunogenesis: (1) constitutional, (2) phagocytic, and (3) lymphatic.

Constitutional immunity is a general all-biological principle that provides all kinds of living organisms with inherent antimicrobial defense. It is not provided by immunoglobulins, but by other mechanisms that develop during ontogeny without induction by any particular parasite or parasitic product. These mechanisms were discovered only recently.

Just 35 years ago, in his handbook on the fundamentals of immunology, Boyd[12] observed that constitutional immunity was a fundamental trait, while expressing regret about the lack of scientific knowledge of this type of resistance. In the years since Boyd's comments, the situation has dramatically changed. Constitutional immunity in relation to many ecological and physiological effects has been revealed and, in many cases, its genetic determinants and molecular tools have been discovered[13,14,15,16,17,18,19].

## *Immunity of Groups, Populations, and Species*

Sheep, mice, guinea pigs, rabbits, and monkeys are susceptible to experimental anthrax, whereas dogs, horses, cats, rats, and pigs are resistant. Herbivores, such as guinea pigs, rabbits, and horses, are highly sensitive to all types of botulinic toxin (Fig.3). Of the carnivores, dogs and cats are highly immune to all types of botulinic toxin. Rhesus monkeys (*Macaca mulatta*) are resistant to type D botulism, but sensitive to the other types[14]. Donkeys, horses, monkeys, and guinea pigs are very susceptible to tetanus, whereas hedgehogs, rabbits, dogs, cats, and chickens possess innate constitutional immunity to this pathogen[14,20]. Rats and mice, in contrast to monkeys, chickens, pigeons, and rabbits, can be affected by diphtheria toxin only after the exposure is increased to 50,000 times the amount that killed guinea pigs. Both rats and mice have natural immunity to diphtheria toxin, although there is no apparent evidence of specific immunoglobulins in their bodies[21].

Such natural immunity is not adequate in all representatives of a species. There is an extraordinary individual diversity in the natural susceptibility of humans to influenza infection. Like any human infectious agent[2,22,23], the influenza virus cannot cause illness in most members of the current world population. Some organisms attacked by influenza viruses fall ill, whereas most individuals display a natural immunity to primary infection. In only a minimal proportion of a human population can the same microbes cause fatal illness.

The Spanish influenza H1N1 pandemic (1918–1919) annihilated between 20–40 million people[24], nearly 1–2% of the worldwide population at the time[25]. Up to 20% escaped death without specific medication and near 80% escaped illness without vaccination through their own innate mechanisms of self-defense (Fig. 4). The 1918 infecting agent was extremely pathogenic, but only for a minority of the world population. The deadly risk from Spanish flu for most people can therefore be assessed as very low.

The rodent species *Meriones meridianus*, which lives on the left bank of the lower river Volga, is 70,000-fold less susceptible to the plague than representatives of the same species that live on the right bank of the same river. In regions of Africa where trypanosomiasis is endemic, indigenous ruminants suffer from mild infections with insignificant morbidity, whereas domestic animals suffer more severely, and recently imported exotic animals suffer usually fatal infection[2].

Genetic factors play a significant role in the incidence and severity of infectious diseases. In a study of an epidemic of tuberculosis in a herd of cattle descended from two bulls, 62% of the descendants of one bull were infected with tuberculosis whereas only 4% of the descendants of the other bull developed the disease[26]. Webster[27] was the first to develop the Mendelian ratios for the inheritance of immunity to microbial infections by mating constitutive immune and constitutive susceptible strains of mice. Through selective breeding, he developed strains of mice that differed in resistance to St. Louis and louping ill flaviviruses. He showed that immunity is inherited as a single, dominant factor.

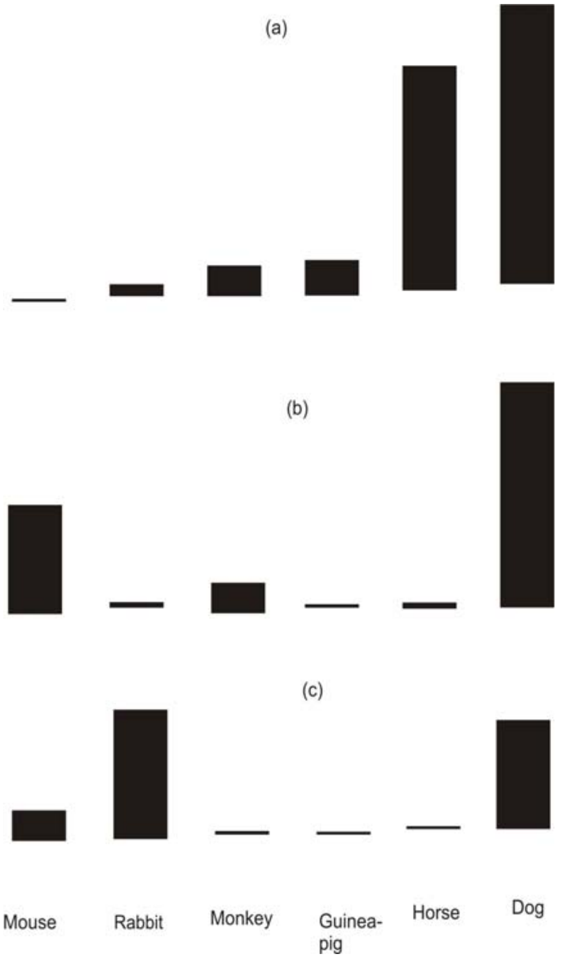

**FIGURE 3.** Variations in the degree of constitutional immunity to (a) anthrax, (b) botulinic toxin, and (c) tetanic toxin in a selection of vertebrata species.

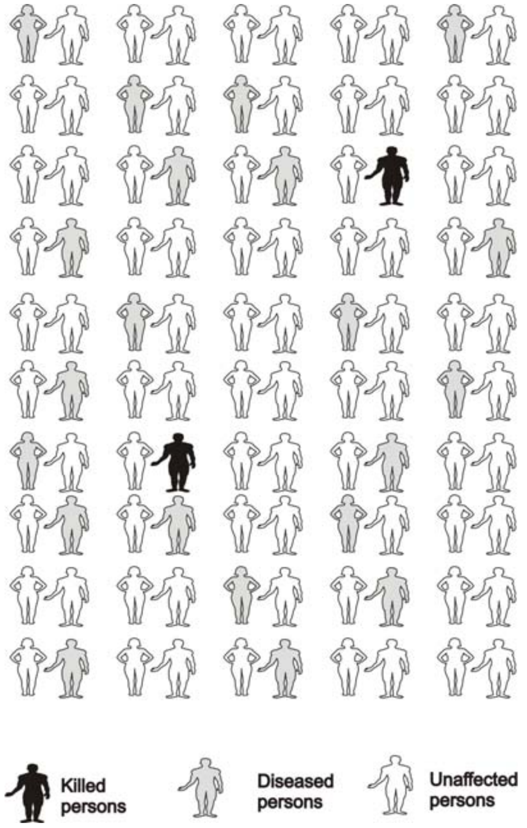

**FIGURE 4**. The Spanish influenza H1N1 pandemic (1918–1919) annihilated nearly 2% of the worldwide population at the time.

Genes that control constitutional immunity to some infections in humans and animals have been identified and mapped to a specific chromosomal location[15,28,29,30,31]. The CCR5 gene 32-base pair deletion provides strong constitutional immunity of human homozygotes to HIV infection[32]; in the heterozygous state, it may provide relative immunity, thus delaying the progression of HIV infection to AIDS in affected individuals[33].

### *Immunity of Cells*

Heterogeneity of cell populations, which are sensitive to an infectious agent, has been reported frequently by many investigators. Sensitivity may involve only a few cells or the majority of cells in a population. When a susceptible cell culture is affected by some microbial pathogenic factor, most cells are killed. At the same time, however, a number of outwardly indistinguishable cells fully preserve their basic functions despite unfavorable conditions.

Cell strains that are spontaneously immune to the lethal effect of diphtheria toxin have been selectively isolated from highly sensitive human cell lines. The toxin-immune cells were found to be 100,000 to 1,000,000 times more resistant to toxin than the toxin-sensitive cells of the parent cell population[21,34]. Thus, the resistance of these cell strains was comparable to the resistance of cells from animal species that are naturally immune to the toxin, such as rat and mouse species. This phenomenon is highly specific; the cell may be immune to microbial agents of one type, but susceptible to another type.

A mammalian cell clone resistant to infection by HIV-1, the murine leukemia virus, and feline immunodeficiency virus has been isolated[35]. The cell clone is genetically recessive for the resistance and, hence, it is lacking a factor that enables infection by retroviruses. The block to infection is early in the virus life cycle, namely at the virus uncoating stage.

## Molecular Make-Up

Pathogenic microbes survive as a result of biosynthetic processes carried out by other organisms. This requires the ability to invade the victim, penetrate its surfaces, reproduce in the victim, interfere with its defenses, and damage the victim. Microbes penetrate organisms and enter cells exclusively through particular molecular processes controlled by specific chemical (molecular ecological) substances (adhesins, toxins, enzymes, cytolysins, or polynucleotides). Infectious disease develops as a result of the interaction between the molecular ecological agents of pathogenic micro-organism on the one hand and the affected body molecular structures on the other. Specificity of host-parasite interaction involves mutual molecular recognition of host and parasite, mediated by chemically complementary molecules.

Harmful micro-organisms usually attack the animal host at the mucosal surfaces of the respiratory, gastrointestinal, or genitourinary tract. Micro-organisms must bind firmly to epithelial cells to avoid being swept away by the secretions that bathe the mucosal surface. In many of the common strategies used by pathogenic micro-organisms to overcome host defense, the first step is the firm adhesion of the micro-organism to the host cell membrane. Adhesion is mediated by glycoprotein molecules called adhesins. Adhesins are the molecular substances produced and used by microbes to adhere to target cells.

Bacterial viruses adhere to the surface of a victim by virtue of certain determinants, which, depending on the species of virus, may be derived from one to ten specific protein molecules. Protein components play a similar role in influenza infection: the so-called hemagglutinins, which function by binding to host cell sialosaccharide receptors. Some viruses, such as the Sendai virus, have surface glycoprotein spikes that adsorb on specific receptors of the cells attacked by the virus. The receptors contain sialic acid.

Microbial adhesion is a highly specific phenomenon. The remarkable specificity exhibited during interaction of microbial adhesins and host cells has been compared with antibody/antigen-specific recognition. A malaria-invasive molecular ecological agent, the 175-kDa erythrocyte-binding superficial protein molecule of *Plasmodium falciparum*, recognizes the terminal Neu5Ac(alpha2-3)Gal-sequences of glycophorin A, a molecular component of the erythrocyte surface[36]. Recognition by malaria molecular ecological agents of sialic acid–containing receptors on the erythrocyte surface might be analogous to influenza virus entry into cells of the victim by means of hemagglutinin, which binds with stereospecificity to sialic acid–capped oligosaccharides. The adhesion of *Escherichia coli* to erythrocytes occurs if the cell surface contains glycophorin Am[37].

Choleraic toxin is able to interact only with ganglioside macromolecules that contain the subunits of ceramide, lactose, galactosamine, galactose, and the radical of sialic acid joined with the molecule of lactose (Fig. 5). But the same toxin does not interact with gangliosides of other types; for instance, those that do not contain galactose or have an additional radical of sialic acid in the end position[38].

Thus, the pathogenic effects of microbes and microbial molecules on molecular targets of the victim are provided by the genetically determined molecular structure of the victim, thereby defining constitutional susceptibility to infection or invasion. As a result of this property of a molecular constitution, viability and propagation of intruders can be restricted or stopped. Constitutional antimicrobial immunity is a complex phenomenon, encompassing several specific and common structures possessed by the attacked organisms and used in defense against the molecular ecological aggression of harmful micro-organisms. These structures are presented with a variety of molecular barriers, such as a lack of specific cell receptors, modification of the specific cell receptor, modification of specific nutrients, lack of a specific nutrient, presence of specific antibiotic and\or poison, and nonspecific mechanisms[19].

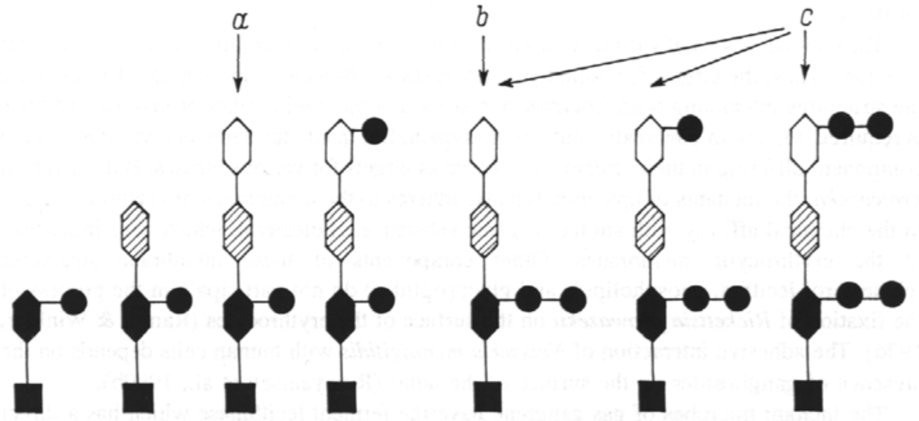

**FIGURE. 5**. Dependence of organism's vulnerability by microbial agents on the presence of gangliosides (1-7) in cell membranes. (a) Choleraic, (b) botulinic, and (c) tetanic toxins.

## *Infections and Biomolecular Evolution*

Certain modifications of an organism's molecular constitution can arise as a result of mutations. The mutations bringing about the origin of similar modifications of the molecular constitution of the victim reduce or even eliminate the efficacy of adequate intermolecular interactions and, accordingly, reduce the success of the microbial aggression. The organisms possessing this modified molecular constitution are incapable of interacting with the microbes; they are not susceptible to the disease and are constitutionally immune to it. Thus, these organisms are not affected by the corresponding microbe but, in contrast, continue to develop normally and produce healthy progeny, while other individuals of the same species become ill and die. On repeated exposure to a given pathogen, the progeny of resistant mutants will finally predominate in a population; an individual protective mutation becomes the property of a group, then of a population, and finally of the species (Fig. 6).

The interactions take place at the levels of specialized molecular structures, peculiar to coactants[2,18]. The biochemical substances are unique for each microbe species. Such molecular-ecological agents of pathogenic microbes are, for example, their polynucleotides (RNA, DNA), proteins, various toxins (including choleraic, tetanic, botulinum, diphteric, etc.), ferments (DNA-ase, RNA-ase, amylase, hialuronidase, sialidase, collagenase, elastase, many other various proteases, sphingomielinase, lecithinase, etc.), surface-active matters, for instance, glycopeptides and glycoproteins, acting as adhesins, hemagglutinins, cytolysins, hemolysins (streptolysin-O, tetanolysin), oligo- and polysaccharides, saccharides, lypoteichoic acid, hydrogen peroxide, etc.[18] The given list of agents is not comprehensive. Each of the enumerated large groups of molecular-ecological agents unites many types of biomolecules highly specific for each microbe species, varieties, and even for each strain.

The targets for these microbe chemical arrows are specific molecular structures in the body of a victim that are mutually complementary and particular in each system. In the interactions with the microbial molecular ecological agents in the bodies of victims, the following take part: polynucleotides (DNA, RNA), oligonucleotides, for instance, nicotinamidadenindinucleotide, split by diphteria toxin; physiologically active proteins, structural proteins including collagen, elastin, various enzymes (for instance, glucoso-6-phosphate-dehydrogenase, alkaline phosphatase, adenylatcyclase, asparaginase, restrictase, translocase, peroxidase, trypsin, other proteases, cholinesterase); immunoglobulins, albumin, phetuin, fibrin, actin, haptoglobins, hemoglobin, ceruloplasmin, various glycopeptides and sialoglycoproteins (glycophorin, mucin); amino acids (f.e. lisine, asparagin); mono-, oligo- and polysaccharides (starch, glycogen, pectin, lignin, cellulose, galactosides, galactosamine-N-acetyl, glucosamine-acetyl, other hexoses, erythritol, sialic acid, sialomucopolysachharides); lipids and fatty acids (lecithin, cholesterol, linoic acid, oleic acid, sphingomielin, phospholipids, ceramide, cerebrosides, gangliosides); metalloorganic compositions (ovotransferrin, ferritin); complement, interferon, vitamins, steroids, colchicine, various aldehydes, ketones, chitons, etc. It should be mentioned that both the molecular arrows of the microbe aggression and the complementary-to-them chemical structures of affected organisms, by their nature and organization, are exclusively various and original for each ecological microbe-victim system[18]. "A particular race of bacteria or virus being adapted to individuals of certain range of biochemical constitutions, while those of other constitutions are relatively resistant"[39].

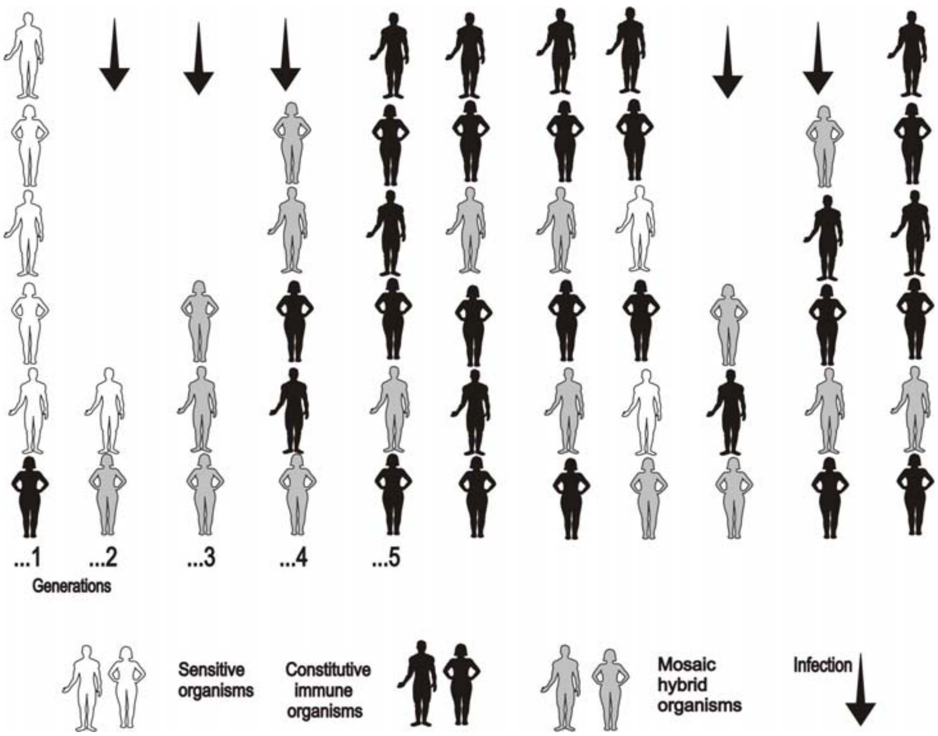

**FIGURE 6**. Evolutionary transformation of immune status of a population in the fight against microbes.

The mutations bringing about the origin of similar stereochemical modifications of the molecular constitution of the victim reduce or even liquidate the safety of adequate intermolecular interactions and, accordingly, reduce the successfulness of the microbial aggression. Such modifications can arise, and really arise in nature due to mutations and other genome transformations that bring about the change of the corresponding biosynthetic processes in the organisms of the potential victims.

The discussed and widely-spread-in-nature group of antagonistic interactions realized by means of biomolecular evolution exerts influence on the viability of organisms involved in these interactions. In addition to that, the action of such molecular-ecological factors on the adequate biomolecular targets can be prevented by the genetically determined peculiarities of the molecular constitution of these targets, arising due to the mutation change peculiar to all the forms of the living matter. Such heritable changes of the molecular anatomy of victims conditioning the inefficiency of the chemico-ecological factors of microbial aggression provide their possessors with genetic (constitutional) immunity to the infection that is baneful to their kinsmen who do not have such constitutional characteristics. Due to this, the possessors of the immune molecular constitution preserve their viability, whereas other individuals perish without producing posterity (Fig. 6).

The biomolecular interrelations proceeding in the microbe-victim systems are of paramount importance for the processes of molecular evolution of living forms and creation of their biodiversity. The molecular factors of microbial aggression have remained, at present, the main biological factors, realizing the natural selection among the people and directing the biological evolution of the mankind[2,18].

Living beings have been subjected to interaction with agents of numerous infectious diseases due to their specific ecological features over billions of years. Species living today presumably possess genes for natural defense against the majority of those diseases[19]. As a consequence, constitutional resistance currently seems to be a leading factor in the immunological structure of populations determining the main parameters of epidemic processes including severity of diseases. In addition, one must accentuate that ecologically different species, subspecies, and ethnic groups have been subjected to interactions with different infectious agents being performed by different changes of their molecular constitution.

## Immune Mosaics

In a population reliant on inherent constitutional immunity to an infectious agent, individuals can be conveniently divided into some categories, as follows (Fig. 7):

1. Totally resistant homozygous organisms that do not contain susceptible structures
2. Mildly susceptible heterozygous (mosaic) organisms, in which a few foci appear and the infection runs a benign course
3. Highly susceptible heterozygous (mosaic) organisms in which the number of susceptible structures is high and the infectious process develops in a severe form with formation of many foci of specific infection
4. Totally susceptible homozygous organisms that contain only susceptible structures

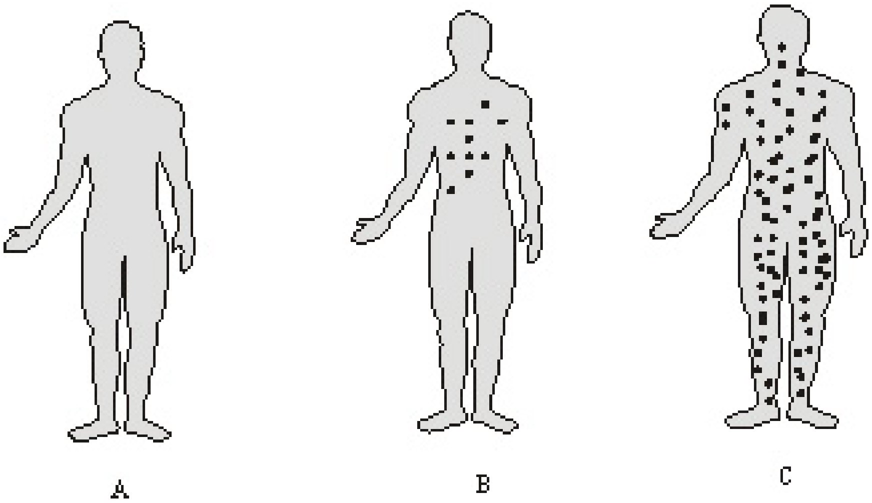

**FIGURE 7**. Individual and intraindividual diversity in the severity of an infectious disease. (A) Totally resistant organism that does not contain susceptible structures, (B) mildly susceptible organism in which a few sensitive foci exists and the disease runs a benign course, (C) organism in which the number of constitutionally susceptible structures is high and the disease develops in a severe form.

## The Origin of Immune Mosaics

The mating of resistant and susceptible individuals gives rise to progeny with intermediate degrees of infectious lesions. These genetically predetermined differences are proof of heterozygosity; the constitutionally immune parent passes on to the descendant a lack of receptivity to a certain infection in some parts of organs, while the susceptible parts are inherited from the second parent. There is no such thing as a hybrid between the two parents.

The clonal histochemical architecture of the small intestine, esophagus, breast, and thyroid was demonstrated in normal animals using a diversity of the X-linked enzyme glucose-6phosphate dehydrogenase (G6PD) as a clonal marker. The results were found to depend on the simultaneous study of tissue from normal mice, mice homozygous for abnormal G6PD levels, and heterozygous mice. A dual population of cells was found in the heterozygous animals in all tissues studied. The esophageal epithelium showed precisely separated, alternating paths of positive and negative cells. Small intestinal crypts were always monophenotypic, whereas villi were polyphenotypic, indicating that crypts are monoclonal and villi derive from more than one crypt. In contrast, thyroid follicles and breast acini showed a mixture of mono- and polyphenotypia, consistent with a polyclonal origin[40]. Thus, it must not be supposed that all parts of the body are equally liable to specific infectious damage.

Each infectious disease expresses at least two categories of the same tissue in the infected organism, outwardly analogous and differing only in their relationship to a given micro-organism. The origin of those mosaic configurations within affected homogenous structures is determined by genetic factors. It can also be by some physical, chemical, or other environmental and physiological influences. In light of the above concept, the origin of individual differences in a course and severity of infectious diseases is becoming better known. This situation emphasizes the importance of further research to determine the respective roles that inbreeding and outbreeding have in the appearance, localization, severity, and clinical course of infectious diseases as well as the new directions of their clinical prognoses and cures.

## Ontogeny of Immune Mosaics

Constitutional immunity may change during ontogeny of the organism. Immunogenic characteristics of the molecular constitution of the organism are controlled by the genetic program of individual development. That is why newborn and 3-week-old mice are totally defenseless against the virus of yellow fever, but by adulthood, they have become completely resistant. Newborn mice are also very susceptible to the herpes virus, but by 4 weeks of age, this sensitivity is replaced by constitutional resistance[41,42]. A general characteristic of most infections in animals and plants is the changed response to disease, which develops in the course of growth and maturation. The changes are particularly dramatic in the neonatal period.

Meningococcal infection presents one of the rare exceptions to this common law. About 15% of all cases of childhood meningitis is due to the meningococcus virus[43]; however, children under 1 month of age rarely fall ill with a meningococcal infection. In a series of 211 cases of neonatal meningitis, *Neisseria meningitidis* was an uncommon organism, with only 2 of the 211 due to *N. meningitidis*[44]. In accordance with these epidemic and clinical observations, cells of human newborns are absolutely resistant to the attachment of meningococci[16]. However, in the neonatal and following periods, the cells become far different and individually sensitive to meningococcal adhesion. Thus, the function of meningococcal receptors on human cells develops during postnatal ontogenesis. In contrast, the influenza viral adhesion is characterized by different individual cell sensitivity in all age groups tested[16]. Erythrocytes of 2-day-old chicks are more sensitive to rabies virus adhesion than those of adult chickens[45].

The constitutional immunity of a cell may change during the ontogeny of the cell. Malarial parasites demonstrate selectivity towards a particular age group of erythrocytes. For example, young mature chicken erythrocytes are selectively attacked by *Plasmodium gallinaceum*. Erythrocytes under 12 days of age are preferentially destroyed by the parasites. The factor involved in such a phenomenon may be that the young erythrocytes contain more lipids in their membrane than the older cells[46]. Age-related changes in resistance of cells to mengo virus and encephalomyocarditis are conditioned by corresponding changes in maturation of the cell membrane molecular composition, namely the structure of the molecular receptor[47].

Senescence increases the frequency and severity of infections, which cannot be explained only by the decline in efficiency of the lymphoid system immunogenic response. The increase in susceptibility of the elderly to certain infectious diseases can be explained by the changes in molecular constitution of organs, tissues, cell clones, cells, and extracellular structures that occur with age. There are developmental changes in the molecular constitution of cells that are correlated with developmental differences in their physiological and ecological characteristic, and this may cause the age-related differences in susceptibility to relevant agents.

## VARIABILITY IN NONINFECTIOUS DISEASES

### Observations

During the last decades of the 20th century, researchers identified bacterial and viral triggers for some diseases that had been considered as noninfectious before[48]. Many bacteria and viruses have been linked to “noninfectious” diseases. Human papillomavirus (HPV) gets an uncontested role in cervical cancer, hepatitis B and C viruses in hepatic cancer, Epstein-Barr virus in lymphoma, schistosomes in bladder cancer, and *Helicobacter pylori* in gastritis and gastric ulcer.

In addition, infectious agents have also been considered as contributors to leukemia, Crohn’s disease, arthritis, and rheumatic fever. However, despite the growing evidence, the role of infectious agents in a wide range of diseases is still contested. For decades, scientists have unsuccessfully attempted to link infection with atherosclerotic cardiovascular disease, as well as to find the link between herpes simplex virus type 1 (HSV1) and Alzheimer’s disease.

The triggering role of microbial agents in autoimmune and psychiatric disorders, as well as in obesity, diabetes, and other metabolic diseases, continues to be controversial[49] and we can continue to consider these groups of disease as noninfectious. From a vast number of diseases, only some of them (atherosclerosis, obesity, senescence, schizophrenia, and autoimmune aggression) will be considered below in an attempt to get a new understanding of this kind of pathology from the viewpoint of biodiversity.

### *Atherosclerosis*

Atherosclerosis, the specific intra-arterial pathological process, is considered to be the world’s biggest killer. Since 1990, more people have died worldwide from only one clinical form of atherosclerosis, coronary artery disease, than any other disease. Cardiovascular diseases now represent more than 30% of all deaths worldwide. Stroke, another clinical form of atherosclerosis, is the second-leading cause of death and a major cause of long-term disability throughout the world[50].

The disease can show itself in different clinical forms from the effect on blood circulation: coronary artery disease (CAD), myocardial infarction, stroke, high blood pressure, or other. Any associations with the above forms have been observed too. The etiology of atherosclerosis remains uncertain. The “response-to-endothelial injury” theory is most widely accepted, but nobody can name which ecological or physiological factor is responsible for both development of this “injury” and its epidemic spread around the world. Racial and other differences in atherosclerosis, i.e., interpopulation, intrapopulation, and age diversity, in the incidence and manifestations of the disease have been discovered in many details. This disease is characteristic of especially variable manifestations.

Individual manifestations and courses of atherosclerosis are highly variable. Many people with structurally advanced atherogenesis may have no symptoms and experience no functional impairment. On the other hand, initially asymptomatic patients may be subjected to sudden cardiac or aortal death, whereas many other people present for a long time with clinically important symptoms and signs of relevant chronic diseases (coronary heart disease, high blood pressure, renal deficiency, or other local dysfunction of blood circulation), or suffer from various consequences of former myocardial infarction or stroke.

Atherosclerosis may begin as early as in childhood, but it is the advanced stages of this condition that become the most dangerous later in life. The earliest pathologic lesions of atherosclerosis continue in the development and further progression of specific patched formations (plaques) on the innermost of arterial wall (Fig. 8), leading to reduced or blocked blood flow (e.g., producing angina pectoris).

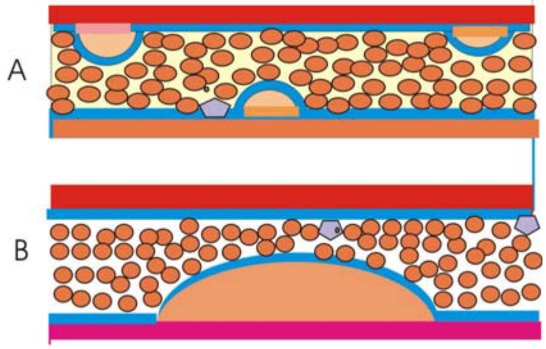

**FIGURE 8**. Some variants of specific patched formations (atherosclerotic plaques) on the innermost of arterial wall.

Although a systemic disease, atherosclerosis manifests in a focal manner and affects stochastically different organs and systems in different patients for reasons that remain unclear. Plaques (either single or multiple) can be individually stochastically localized on the wall of either one or more arterial vessels. Plaque buildup in the vessels that supply an organ with blood may cause relevant dysfunctions of that organ. Symptoms of any atherosclerotic affection depend on the size of the affection's location as well as on the danger of the focal bloodstream blockage. The focal character of the affection is the principal feature in this kind of pathology. According to location and degree of specific affections, this can result in relevant local ischemic accidents that manifest appropriate clinical syndromes and outcomes[50].

Coronary (heart) artery atherosclerosis leads to coronary artery disease. Thoracal aorta atherosclerosis leads to an aortic aneurism. Atheroma in arm or leg arteries may cause occlusive disease of the relevant peripheral artery. In carotid arteries of the brain, atherosclerosis leads to sudden dizziness, weakness, loss of hearing or speech, and blindness. Cerebral artery atherosclerosis may lead to a stroke on one side of the body. Hepatic artery atherosclerosis leads to insufficiency of the relevant lobe of the liver. Affection of the pancreatic artery leads to insufficiency of the pancreas. Abdominal aorta atherosclerosis leads to an abdominal aortic aneurism. The location of plaques in a mesenterial artery can induce ischemic bowel disease. Atherosclerosis of a renal artery can lead to insufficiency of the relevant kidney, for instance, high blood pressure resistant to treatment. Peripheral artery atherosclerosis is caused by plaque buildup in the arteries that supply the extremities of the body with blood (such as the hands and feet). In the femoral artery of the legs, atherosclerosis leads to cramping and fatigue in the calves of the legs when walking and, finally, to necrosis of the legs or fingers.

## Obesity

The current global epidemic of obesity is associated closely with a plethora of dangerous chronic illnesses, such as heart disease, diabetes, high blood pressure, stroke, different kinds of cancer, liver disease, gout, disease of the joints, etc. Obesity is now the second-leading cause of excess deaths in the U.S.[51] and may reduce the life expectancy for the most affected populations[52].

The shape, location, size, and rate of obesity progression may be different in different individuals or different organs as well. Equally, with total affection of almost all the body, there are several key areas of restricted focal fat-storehouse locations around the body. The areas of a human body where solitary focal obesity can be observed include facial jowls, neck, upper back of the arms, upper back underneath the bra straps, areas near the underarms, waist, love handles, hips, breast, buttocks, abdomen, belly, thighs, knees, calves, and ankles. In central obesity (or "apple-shaped'" or "masculine" obesity), the main deposits of body fat are localized around the abdomen and the upper body. In mild and intermediate forms of the disease, total affection of almost all the body can include all the locations, whereas the locations in the form of solitary bulges also can be observed in various individual combinations. In most cases, obesity is presented by focal manifestations of individually different grade.

The locations and sizes of fat storehouses are different for each individual (Fig. 9). Someone may have only one small bulge, whereas another person may have three or four local areas where excess fat exists. The neck alone can also be composed of an excess amount of fat. Some people may have sagging jowls right underneath their jaw line. The whole belly or its upper and lower parts are the typical areas where extra fat is stored. A couple of folds occur when increased fat accumulates in the upper back. Some people have bulges of fat that begin below the underarm and then come down before the waist starts. Some affected women have fat in the back of the upper arms. The thigh, especially its outer part, is very commonly affected. Some patients have a significant amount of fat in the inner low quadrants and not as much in the outer areas of the buttocks. For some people, the fat is stored in the upper buttock, whereas for others, it is in the lower outer buttock. Extra fat bulges may be symmetric or asymmetric. Someone can be heavier on the right side, but another on the left. Not much fat has been observed in the calf or ankle. Fatty calves and ankles are often an innate condition that patients have had from childhood, even when they have no weight problems. The sizes of obese masses and their dislocation around the body predestine individual diversity in the course and severity of obesity.

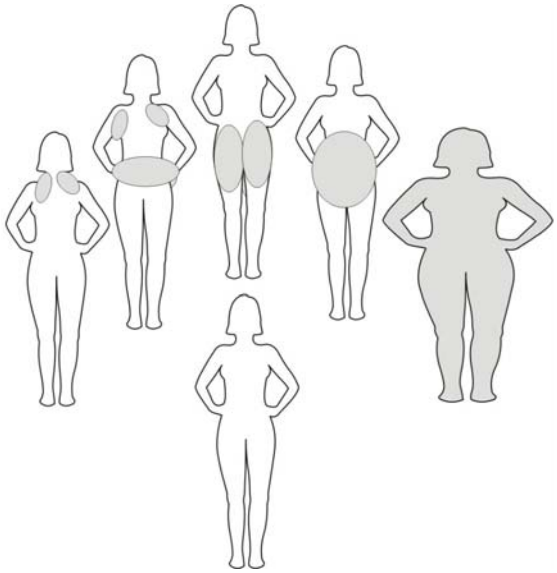

**FIGURE 9**. Some individual variants of the sizes and locations of the fat storehouses in obesity.

Thus, one can conclude that within every obese human body, there are at least two coexisting parts of fat tissue with unequal predisposition to obesity. The parts exist in a form of separate patches of different sizes and locations, and are stochastically dispersed around the body. The reasons for some original dislocation of fat have not discussed until recently[49].

In contrast, the genesis of abnormal fat metabolism has been discovered up to a molecular level. Every kind of living being is provided with a constitutive physiological system that maintains normal body structure within a genetically predetermined shape and size. A part of the physiological system that regulates body fat has now been[53]. It acts through the molecular physiological mediators of energy intake and energy expenditure. The ability to burn fat for energy and the achievement of satiety are performed by the functioning of a particular set of hormones and other molecular physiological agents. The system includes the hormonal regulation of food intake, first by leptin and insulin. In a case of obesity, the work of this precise and powerful system becomes disturbed.

In a normal organism, leptin performs the role of inhibiting excess food intake[54]. Disturbance of the leptin production or function determines the development of obesity. Deficiency of leptin production results in an insatiable appetite, leading to severe early-onset obesity. The same result is achieved by a mutant modification of the leptin structure that forms incongruence between leptin and its receptor. Both states can be corrected by leptin treatment[55].

On the other hand, in an obese body, the leptin function can be normal or its content increased by leptin treatment, but its performance can be stifled by a derangement of receptor cells, i.e., by constitutional immunity fat cells to leptin’s influence because of incongruent receptors[49,55,56].

Analogous dramatic immunity of cells to specific action of relevant physiological agents functions in many kinds of hormonal pathology[49]. For example, the immunity of cells to insulin is a major determinant of the decline of glucose tolerance. Similarly, noninsulin-dependent diabetes mellitus is characterized by pathological hyperglycemia in the presence of higher-than-normal levels of plasma insulin. Likewise, a pathogenic decrease in cell sensitivity to vitamin D3 determines the familiar forms of rachitic, and the immunity of cells to androgens causes the phenomenon of testicular feminization. An analogous resistance of cells to corticosteroids determines the pathogenesis of Cushing’s disease. In yet another example, constitutive immunity of cells to thyroid hormones determines the genesis of a range of relevant disturbances (RTH-syndrome). This resistance is an inherited inability to respond appropriately to the T3 hormone linked to mutations in the thyroid hormone receptor (TR)-beta[57]. The principles of cell immunity to physiological agents are analogous to those in the constitutional (genetic) immunity to infections[17,49].

There are some commonalities in the structures and functions of molecular physiological and infectious agents[2,17,49]. The B chain of the cholera toxin and the beta subunits of thyrotropin, luteinizing hormone, human chorionic gonadotropin, and follicle-stimulating hormone are shown to have a region of sequence analogy believed to correlate with their ability to bind to receptors on cell membranes. A sequence analogy is also defined in the alpha subunits of these glycoprotein hormones and the region of the cholera toxin A1 chain believed to be responsible for adenylate cyclase activation[58]. Like hormones, the cholera toxin increases adenylate cyclase activity, raising levels of cyclic adenosine 3',5'-monophosphate, which activates a cellular lipase[59].

What is more, many hormones share their specific targets with the acting molecules of infectious agents. Tetanus toxin binds specifically with the thyroid thyrotropin receptor[60]. Similarly, the rabies virus receptor appears to be closely associated with the acetylcholine receptor. Epstein-Barr virus has a strong tropism for lymphocytes and those of the complement C3 receptor[61]. The human T-cell leukemia virus preferentially infects the cells of the helper/inducer class[62]. Likewise, the cardioviruses and influenza A and B viruses bind to the glycophorin A molecule on human erythrocytes and this glycoprotein also carries the M and N blood group antigens[63]. Virus-induced obesity in mice alters their response to leptin[64]. Human adenoviruses increase adiposity in humans[65] and other animal models[66,67].

Taken together, these data make it permissible to suppose that, in fact, all “viral”- and “bacterial”-specific receptors have evolved to interact with microbial pathogens, but to provide the physiological functions of hormones or other physiologically active molecules. The evolutionary adaptation of harmful microbes to a parasitic way of life could be performed by the exploitation of pre-existent physiological receptors or other cell structures and functions[65]. The subsequent evolution and spread of genetic immunity to infections (Fig. 6) could create a constitutional genetic immunity to the molecular physiological agents responsible for regulation of fat intake and metabolism, thus providing the spread of relevant forms of pathology[49].

Nevertheless, although the spread of obesity is progressing as in a worldwide pandemic, the possibility that infections might be etiologic agents in obesity has received relatively little confirmation. Experiments link obesity to four animal viruses (rabies virus, Rous-associated virus, Borna virus, and SMAM-1). A human virus, adenovirus 36, increases adiposity in experimentally infected chickens, mice, and marmosets, but direct correlation of human obesity with an epidemic of any infection was not found[48,65,66,67,68,69]. Nevertheless, the indirect action of infections on obesity genesis and spread cannot be denied today[49].

On other hand, the mutant formation of the life-saving genetic immunity to infection could create a life-threatening resistance to the relevant hormone. This kind of individual molecular constitution could spread in a population as a result of natural selection for genetic immunity to infections that has performed differently in ecologically distinct populations[18,19 ], thus forming ethnic differences in the molecular constitution of both hormones and their targets. Thus, molecular physiological agents of one ethnic group become xenobiotic to the analogous agents of another group, and vice versa[49]. On the other hand, any change in one molecular physiological agent should entail relevant evolutionary changes in all other molecules connected to it[18]. Thus, the evolutionary process removes the molecular physiological incongruence within an ethnic group, but creates an incongruence between groups.

Humans are extraordinarily diverse in their manifestations of obesity. Affected people have many differences in manifestations of their obesity as well as in the grade of its expression.

Thus, one can conclude that focality of affection is the principal feature of obesity.

The origin of this very important feature has been ignored by previous systematic investigations. Heretofore, the origin of individual and intraindividual differences in location, shape, and size of fat storehouses has formed an unsolved riddle of obesity. Should one suppose that the physiological functions of fat metabolism and their regulation in obesity are disturbed differently in different populations, in different generations, as well as in different body areas of the same person?

Different parts of a body can become obese at different ages. The question arises immediately about genetic determination of individuals and intraindividual diversity in the age of obesity development. How can we account for the fact of individual differences in the age of obesity development? Should one accept that, in some individuals, the genes of obesity begin to work in the period of childhood, whereas in other bodies, the time to become obese begins at adulthood? Can one suppose that some human genomes contain different genes of obesity that work at different ages? Many observations about constitutional immunity to infectious agents and senescence confirm that correlation of sensitive and constitutionally immune cell clones of an organism can change with age[2,16,70] and allow us to accept this working hypothesis for the discussed cases of analogous immunity to leptin or other molecular physiological agents involved in the development of obesity.

How can we account for the fact that the analogous cells of an organism differ from one another when all contain the same genome complex? Up to the end of the 20th century, it was a riddle for both pathology and genetics[1,49,70].

Although it is now an obvious truism that a person's genetic makeup has a principal influence on the development of obesity, the special characteristics of the genome that determine the zonal distribution of obesity around the body was unknown until the beginning of 21st century[49]. In contrast, the origins of the analogous features of infectious diseases and some noninfectious ones were deciphered earlier, at the end of 20th century[1,2,70,71].

## Senescence

Senescence is one of the most important topics of human biology and medicine. It is the result of a productive form of aging, leading to organism's programmed death. In analyzing the origin of senescence, the entire body of facts is considered. Scientists are using observations in populations and clinic, cells in culture, yeast, nematodes, and mice, and the human genome to gain a better understanding of why and how we age. Although the aging field is full of theories, the phenomenon continues to remain mysterious. The pages immediately following are devoted to what we have to say that is new about the phenomenon from the viewpoint of constitutional immunity. An attempt was made to analyze the nature and importance of this kind of human body genetic diversity, using examples from epidemiology, clinical observations, genetics, immunology, and molecular biology.

The human life span has varied historically from generation to generation, from population to population, and from individual to individual. Senescence changes are not synchronous in any population. Different people do not grow old simultaneously. If we look at the current diapason of individual life spans, we would see a definite contrast between them. Jeanne Calment was the oldest known person whose exceptionally long life span had been verified by official document. She died at 122 in 1997[72].

In contrast, there are premature aging syndromes (Werner Syndrome, Hutchinson-Gilford Progeria Syndrome, and some others) that mimic many signs associated with aging and result in very early death. For example, Hutchinson-Gilford Progeria Syndrome is a genetic state that accelerates the aging process to about seven times the median rate or about seven times the life span of Jeanne Calment. This kind of human phenotype, of very short life span, is associated with the early onset of the usual diseases of aging. Because of this accelerated aging, a child of 10 years will have similar respiratory, cardiovascular, and arthritic conditions that a 70-year-old adult would have. If progerics reproduce, their offspring would be expected to inherit the same genetic status[73].

Modern human populations are very different in their age structure as well as in intensity of their senescence. The elderly may make up only the smallest part of a population, for instance, in Cuwait (Kuwait), or a far larger part of it, for instance, in Monaco (Fig. 10).

It has been believed for a long time that our senescence is programmed into our genes and it is true. First, genetic regulation determined the essential life span of every species. Second, findings from animal studies have provided evidence that individual genes can have a significant effect on life span[74]. Third, there are genetic differences between human populations and families. According to Huang et al.[75], Werner's gene (WRN) is a longevity-assurance gene. If WRN is missing, then we see accelerated aging[73]. Furthermore, common polymorphisms in some genes influence life span, probably mainly through their association with diseases[76] that arose with the development of human society[70]. The data and conclusions are confirmed by observation of individual variations in the manifestation of senescence.

When any individual begins to grow old, only some places in his/her body are involved in the process. The entire human body does not undergo the senile regression simultaneously[77]. The forms and rate of aging progression are different in different species, individuals, organs, systems, tissues, organelles, and macromolecules. At the same time, individually, no tissue, organ, or system becomes functionally exhausted even in a very old organism[78]. That means that while some parts of a considered organ are affected by senescence and exhausted, others continue to function. They have a longer life span.

All tissues can undergo regressive structural changes with age, but not simultaneously and not uniformly in their different locations, sizes, and dispersion. Different parts of the entire human body age differently. Moreover, the forms and rate of aging progression are usually different, not only in different organs, but also in different parts of the same organ. Also, they may be different not only in different tissues, but in different places of the same tissue. For instance, in one person, the senile wrinkles may be displayed mainly on the face, but in another person, they may be located mainly on the back of his neck. When the smooth skin of the face turns into wrinkles, it happens just owing to the local inequality of senile regression. At the same time, however, a number of neighboring parts of the skin fully preserve their basic structure and function.

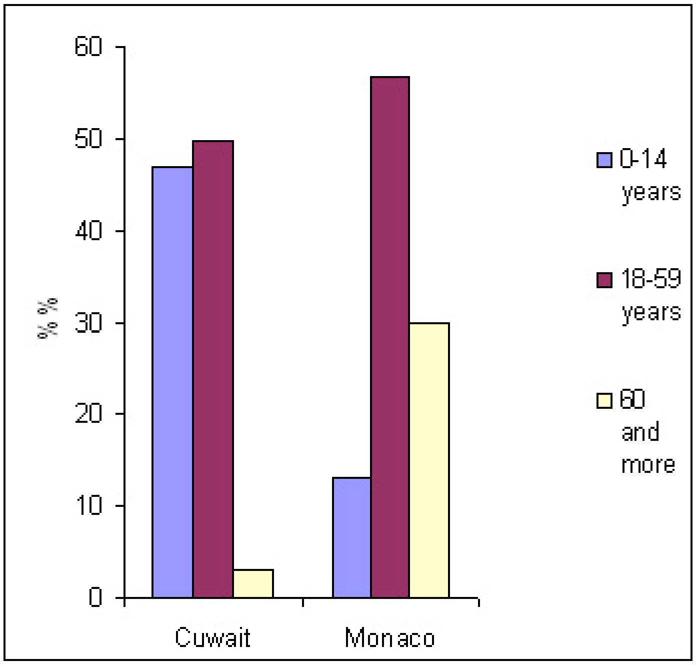

**FIGURE 10**. Different age structure of two human populations.

Another of the most evident examples of such senescence inequality is the difference in the rate and distribution of hair turning gray. Some people can begin to gray before age 30. Most people over 40 have some graying of scalp hair. Although the tendency to graying is inherited, as is baldness[79], different parts of the hair may regress at different times and may even be located in close proximity to unaffected loci of the same skin. In the early steps of senescence, gray hair flecks may be observed on the temple (Fig. 11, left) or in the beard (Fig. 11, right). Usually they are located symmetrically, being surrounded by normally colored hair. Asymmetrical location, both of solitary and multiple gray hair flecks, also can be observed. In contrast, in the late steps of senescence, a plethora of gray hair surrounds the dispersed flecks of normally colored hair.

Different places of an entire organ or tissue may be affected by senescence at different ages. Thus, although aging is usually named as a generalized process, in fact, aging is a diffuse process with stochastically disseminated focal manifestations. Every senile affection is dispersed in disseminated focal areas of the aged organism. Without any exception, a relevant tissue is affected irregularly and duration of life is determined not only by a degree of senescence dissemination, but in some cases, by location of relevant changes. This is a widely known phenomenon to which special research attention has not been called before. The nature of dapple aging manifestation has completely eluded investigation and interpretation.

ne could suppose that those foci do not appear simultaneously, but according to their own programs of aging. The early aged foci may form in only small solitary places of a tissue, an organ, a system, a region, or an organism or in the large areas of it. One can suppose that the proportions and the features of dislocation change according to features of individual genetic makeup and thus determine individual variations in the course of senescence as well as in the life span of an organism under consideration.

Senile regression reveals heterogeneity patterns in cell clones too. Cells also age differently. There is now *in situ* evidence that senescent cells accumulate with age in human tissue. For instance, the share of fibroblasts that express a marker of senescent cells are readily apparent in the dermis of skin from old donors, but much less so in skin from young donors[80]. Recent studies on longevity of organelles and molecules also indicate a large range of lifetimes[81,82]. Such local sensitivity to senescence revealed by tissues, cells, and subcellular and molecular structures may involve only a few parts of the body or a majority of them.

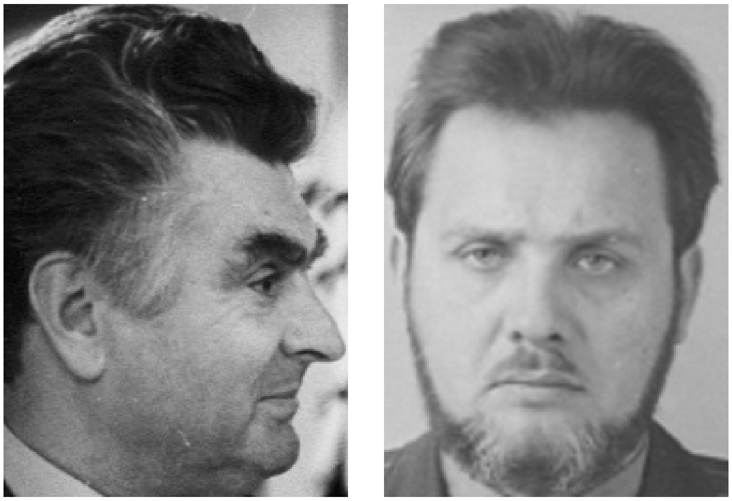

**FIGURE 11.** Some individual variants of dapple location of gray hair flecks in a temple (left) and a beard (right).

In all cases, a considered body consists of parts that are not predisposed to early age, along with those parts that are susceptible to senile regression and, thus, have grown old. One can conclude that within every human body, there are coexisting pools (clones) of homogeneous cells and subcellular structures with unequal predisposition to aging. The quantity, dispersion, and localization of more susceptible structures are individually diverse and variable. It is the reason for the organism's varied individual predisposition to aging in respect of its course, degree, and time of development. Thus, one may assume that different parts of a human body can have unequal limit of life duration; each of them with its own "biological watch" telling a different time.

Thus, one of the most characteristic and widely observable features of senescence, usually ignored by scientific investigation, is very variable dapple distribution of its manifestations seen within systems, organs, and tissues of the same individual.

Cultured cells from people with hereditary premature aging syndromes grow old prematurely. One can suppose that the mosaic dapple dispersion of senile changes is also caused by different local predisposition of some parts of outwardly homogenous tissue to senile regression. The focal resistance to aging can explain why any parts of a tissue are affected by the process, while at the same time, the morphologically analogous components of the same organism remain uninvolved, although both exist under the same conditions and may be equally attacked by relevant agents. These morphologically analogous parts, which vary in resistance/sensitivity to aging, appear as a nidus, and the distribution of such foci are distinct for different people.

## *Schizophrenia*

Schizophrenia is a devastating psychiatric disorder characterized mainly either by "positive symptoms" that include delusions and hallucinations, or "negative symptoms" that include blunted emotions and social isolation, and cognitive deficits that include impairments in executive function, attention, and working memory[83]. The mechanisms of schizophrenia development are considered as a mosaic of multiple endogenous factors including changes in the biochemistry of the brain function, changes in the structure of the brain, and genetic predilection. A possible role of viral infections and head injuries are also discussed. Finally, schizophrenia is considered as a group of related diseases, some of which are caused by one factor and some by another[84].

Although schizophrenia occurs worldwide at an average frequency of about 1% (i.e., nearly 65,000,000 around the world), there is about tenfold interpopulation variation in its prevalence[85]. The lower frequencies of schizophrenia are seen in “nonindustrialized” naïve societies. The “industrial” societies have many more chronic schizophrenics. The positive correlation between the “complexity” of a society and the number of schizophrenics it allows has been revealed[86]. The aboriginal Taiwanese groups are found to have much lower rates of schizophrenia than those found in an ethnic Chinese population also living on the same island[87].

The highest rates for schizophrenia are characteristic of the more mixed populations. In the state of Hawaii, 1,689 schizophrenic patients were followed from 1962–1972. Those of Japanese and Chinese descent had the highest rates for schizophrenia, those of Philippine descent had intermediate rates, and Caucasians had the lowest[88].

The disorder is more prevalent in families where schizophrenia has been diagnosed previously. The bench psychiatrists feel justified in strongly suspecting that our species is divided into a very large majority of individuals who do not possess the schizophrenic genotype, and a small minority (perhaps 5%) who do[86].

The schizophrenic patients demonstrated very marked variations of course and outcome of the disease. In some cases, schizophrenia may develop very gradually. A gradual buildup of symptoms may lead to an acute or crisis episode of schizophrenia. Sometimes, schizophrenia has a rapid or sudden onset with very dramatic changes in behavior that occur over a few weeks or even a few days. Some people have very few such attacks in a lifetime; others have more. Some people lead relatively normal lives between episodes. Others find that they are very listless, depressed, and unable to function well. In addition, cases of chronic schizophrenia are known.

The individual manifestations of schizophrenia are extraordinarily diverse. Initial signs of the disease onset include over 20 very impressive manifestations, such as excessive fatigue and sleepiness or an inability to sleep, inability to concentrate or cope with minor problems, dropping out of activities (skipping classes), decline in academic and athletic performance, deterioration of personal hygiene, eccentric dress, forgetfulness, apparent indifference (even in highly important situations), bizarre behavior, frequent moves or trips or long walks leading nowhere, excessive writing without apparent meaning, inappropriate laughter, peculiar use of words or language structure, strange posturing, staring, vagueness, social withdrawal, isolation and reclusion, deterioration of social relationships, undue preoccupation with spiritual or religious matters, drug or alcohol abuse, unusual sensitivity to noise or light, low tolerance to irritation, inability to express emotion, irrational statements, conversation that seems deep, but is not logical or coherent.

The development of schizophrenia does not annihilate the initial abundance of manifestations, but makes it more expressive and diverse. The initial signs discussed above begin to be supplemented by some symptoms including delusions (false beliefs), paranoia, hallucinations (hearing or seeing things), extremely disconnected speech or thinking, lack of speech, lack of feelings, feelings that do not match the situation, social isolation, inability to function (at home, work, or school), lack of interest or initiative, lack of energy, problems with hygiene or grooming, odd behavior, overreactive senses, pacing, rocking, catatonia (being unable to move) or very disorganized behavior, strong feelings of depression, anxiety, anger, or unreality, excessive concerns about one's body.

Each of the above manifestations, both initial and those that develop, can appear either alone or in association with any quantity of any other signs of the disease. This indicates that very different parts of the mind can be involved, whereas the neighboring ones continue to function normally; although before the onset of the dysfunction, both the affected and unaffected parts of the brain had equal chances to be damaged. One can propose this as a result of unequal constitutional susceptibility of the discussed structures and their functions to relevant etiologic agents responsible for onset of the disease.

The mind function of the human brain consists of a myriad of transmissions performed inside and between neurons by a plethora of specialized biomolecules. In the brain afflicted with schizophrenia, something goes wrong in this system of communications between nerve cells. Schizophrenia is associated with dysfunction of glutamatergic neurotransmission, namely with abnormal glutamate receptor-related intracellular signaling in the thalamus. Given the complexity of the glutamate neurotransmitter system, there are many locations where disruption of normal signaling could occur and give rise to abnormal glutamatergic neurotransmission in schizophrenia. There is a family of intracellular molecules enriched in the postsynaptic density that target glutamate receptors to the synaptic membrane, modulate receptor activity, and moderate glutamate receptor-related signal transduction[89].Schizophrenia is a genetically complex disorder, making the identification of susceptibility genes formidable[90]. Schizophrenia-susceptibile genes converge on synapses, glutamate, and NMDA receptors[91].

Molecular origin of focal character of schizophrenic dysfunctions has been confirmed by experiments with special neuroplegic chemicals. Individual humans are known to differ genetically with respect to amphetamine sensitivity and other dopamine agonists[92]. Increased sensitivity to amphetamines is characteristic of schizophrenics and tests have been suggested to identify those individuals who possess the schizophrenia genotype[86]. In patients with schizophrenia, D2 dopamine receptors in the basal ganglia have been observed to be more numerous than in patients with no history of neurological or psychiatric disease. It has been suggested that this increase in the number of receptors is a result of a biochemical abnormality intrinsic to schizophrenia[93].

Amid the future findings on the etiology of schizophrenia, one can predict the disclosure of the phenomenon of functional cytoecological dysfunction, dependent on constitutional incongruence between molecular neurotransmitters and their receptors possessed by outwardly homogeneous clones of different molecular phenotypes, being distinguished by tiny, but very important, features of their molecular constitution[49].

## *“Autoimmune” Diseases*

This group of diseases unites various destructive inflammatory processes that damage or even eliminate some parts of the affected body. It is acceptable to *suppose* that those diseases occur when some parts of a body became able to be damaged by the body’s own lymphatic system of reactive immunogenesis. The ability of the system to recognize some of its own constituent parts (down to the submolecular levels) as "nonself" results in its response against some of the body’s own cells and tissues. Unfortunately, this theory is unable to interpret the origin of extraordinary differences in the course and severity that is characteristic of all diseases of this category.

There are important interpopulation differences in the prevalence of different kinds of autoimmune aggression. Incidence rates vary among them, from less than one newly-diagnosed case of systemic sclerosis to more than 20 cases of adult-onset rheumatoid arthritis per 100,000 person-years. Prevalence rates range from less than 5/100,000 (e.g., chronic active hepatitis, uveitis) to more than 500/100,000 (Graves’ disease, rheumatoid arthritis, thyroiditis)[94].

In spite of accepted etiological commonality, there are also important individual (intrapopulation) differences between various localizations of autoimmune aggression; e.g., 5% of the U.S. population is affected by autoimmune diseases, the other 95% is not affected. Autoimmune diseases are more common among women and often increase in incidence following reproductive years[95]. At least 85% of thyroiditis, systemic sclerosis, systemic lupus erythematosus, and Sjögren disease patients are female[94]..

Although most autoimmune diseases can occur at any age, some diseases primarily occur in childhood and adolescence (e.g., type 1 diabetes), in the mid-adult years (e.g., myasthenia gravis, multiple sclerosis), or among older adults (e.g., rheumatoid arthritis, primary systemic vasculitis)[94].

Autimmunity can affect either blood (autoimmune hemolytic anemia, pernicious anemia), blood vessels (polyarteritis nodosa, Wegener's granulomatosis), digestive tract (autoimmune hepatitis, Behçet's disease, Crohn's disease, primary bilary cirrhosis, ulcerative colitis), eyes (Sjögren's syndrome, uveitis), glands (Graves' disease, thyroiditis, type 1 diabetes mellitus), heart (myocarditis, rheumatic fever), joints (ankylosing spondylitis, rheumatoid arthritis), kidneys (glomerulonephritis), lungs (sarcoidosis, scleroderma), muscles (dermatomyositis, myasthenia gravis, polymyositis), nerves and brain (Guillain-Barre syndrome, multiple sclerosis), or skin (pemphigus, psoriasis, scleroderma) separately or in association with other organs.

The manifestations of discussed diseases are extraordinarily diverse. This depends both on which tissue of the body and which part of the body is targeted by the relevant etiologic agent. Multiple sclerosis and Guillain-Barre syndrome (MS and GBS) are briefly considered below to illustrate the clinical diversity of this kind of human pathology.

## Multiple Sclerosis (MS)

MS arises as a result of a chronic inflammatory demyelinating process that damages or eliminates the protective myelin sheath that surrounds the nerve fibers in the central nervous system (CNS) (the brain, spinal cord, and optic nerves). This damage or loss of myelin can block or slow the conduction of nerve impulses in the affected areas of the CNS, causing local disruptions in the signals sent through the nerve fibers to and from the brain. The myelin can be damaged or eliminated in multiple areas of individually very different locations and sizes. The initial cause of MS is unknown; e.g., it is hypothesized that a viral infection starts a process in which the lymphatic immunogenic system becomes able to attack the body’s own myelin by specific antibodies that begin to elaborate after pathogenic triggering.

The genetic component of MS pathogenesis seems to be undoubted. MS is characterized by a high prevalence in European populations and those of European descent, and lower risks for other ethnic groups. The rarity of MS among Samis, Turkmen, Uzbeks, Kazakhs, Kyrgyzis, native Siberians, North and South Amerindians, Chinese, Japanese, African blacks and New Zealand Maoris, as well as the high risk among Sardinians, Parsis, and Palestinians, clearly indicates that the different susceptibilities of distinct racial and ethnic groups are an important determinant of the uneven geographic distribution of the disease[96]. Nevertheless, nobody has found chromosomal structures able to influence susceptibility to MS. For instance, no evidence has been found for association of a European-specific chromosome 17 inversion with MS[97].

Although genetic epidemiology studies have shown an increased familial aggregation of cases, intrafamilial concordance in clinically affected regions, age of onset, or clinical evolution has not been found[98].

The areas of demyelination and inflammatory changes in the CNS are multifocal and the symptoms of the disease vary according to which parts of the CNS are damaged by inflammation and the destruction of myelin. The most common early symptoms of MS may include muscle symptoms (muscle weakness, leg dragging, stiffness, a tendency to drop things, a feeling of heaviness, clumsiness, or a lack of coordination) and visual symptoms (blurred, foggy, or hazy vision; eyeball pain, especially with movement; blindness; or double vision). Optic neuritis (a sudden loss of vision and eye pain) is a fairly common initial symptom, occurring in up to 23% of those who develop MS[99].

Sensory symptoms (tingling, a pins-and-needles sensation, numbness, a band-like tightness around the trunk or limbs, or electrical sensations moving down the back and limbs) and balance symptoms (light headedness or dizziness, and a spinning feeling [vertigo]) are less common at the beginning of the disease. Sudden sensorineural hearing loss is rarely the first manifestation of MS, but in a case of a 31-year-old woman, sudden hearing loss in the left ear was the first monosymptomatic manifestation of the disease[100]. As MS progresses, symptoms may be added by spasticity (stiff movement), tremors, pain, difficulty controlling urination, depression, and difficulty thinking clearly (cognitive impairment). All above-mentioned symptoms may appear either alone or in any thinkable combination. Any associations of the symptoms can be observed. That is why the manifestations of MS can vary widely from one person to another, and no two cases are alike.

## Guillain-Barre Syndrome (GBS)

Like MS, GBS is an inflammatory demyelinating process that attacks the protective myelin sheath that surrounds the nerve fibers in peripheral nerves. It is thought that this process is triggered by a viral infection that predisposes a body to produce relevant demyelinating autoantibodies. The damage or loss of myelin can block or slow the conduction of nerve impulses in the affected areas of peripheral nerves, causing local disruption in the signals sent through the nerve fibers to and from relevant organs, resulting in their dysfunction (outside of the brain and spinal cord), muscle weakness, and sometimes paralysis.

The areas of demyelination and inflammatory changes can be either solitary or multifocal, and the symptoms of the disease vary individually according to which parts of the peripheral nervous system (PNS) are damaged by inflammation and destruction of myelin. These findings suggest the changing distribution of the demyelinating lesions and a different reaction to the unknown pathogens in different parts of a body.

GBS occurs throughout the world, with a median incidence of 1.3 cases per 100,000 population (range, 0.4–4.0). The disease can affect people of both sexes and all ages, and has been reported in all races, although in different prevalence. Males are more commonly affected than females, and there are peaks in young adults and the elderly. GBS is a leading cause of acute neuromuscular weakness in the developed world. Mortality rates vary widely. Prevalence of the disease in developed countries is higher than in developing ones[101].

Pain (usually in back, hips, legs) and paresthesias are characteristic of most cases as well as autonomic dysfunction, transient hypertension or hypotension, sinus tachycardia, urinary retention, and ileus. Tendon reflex loss is a common early symptom that starts with the ankles and progresses proximally. Mild sensory loss includes vibratory and proprioception. Weakness is observed most often in the legs; symmetric, proximal, and distal, and progressive in severity. Cranial nerve weakness is also symmetric, the function of the tongue is affected most commonly. Most patients reach a nadir in 2–4 weeks; about one-third of patients become bed-bound, one-third require ventilatory support secondary to respiratory muscle weakness. The patients may be chair- or bed-bound, may need artificial ventilation, and frequently experience dysautonomic dysfunction; 5–15% of the patients die and more patients are left with a disabling motor deficit and/or fatigue[102].

Unlike MS, GBS and its variants (the Miller Fisher syndrome, chronic inflammatory demyelinating neuropathy) have conventionally been considered diseases exclusively of the PNS. In the last decades, however, several reports of CNS involvement in peripheral neuropathy have challenged this view. Apart from the classical features, the patients also presented with reversible loss of visual acuity, suggesting CNS involvement[103]. In addition, acoustic nerve conduction abnormalities can be observed in GBS. Hearing improved during early convalescence[104].

Mild GBS has been diagnosed in 12 (4.7%) of 254 patients with various degrees of GBS affection. All of these 12 patients were able to walk throughout their illness; 10 had paresthesias, 7 had prominent pain, and 9 had ataxia. They took 8 days, on average, to reach their maximal degree of weakness. Thus, cases of mild GBS reach a clinical nadir in a similar time to those with more severe disease[105].

Like MS, GBS may have a relapsing course. In several large series of Caucasian cases, the recurrence rate was estimated to be 1–10% of GBS patients after complete recovery. In 76 Japanese cases, the recurrence rate was estimated as 4%[106]. Of the 220 patients of acute idiopathic demyelinating polyneuritis seen over a 7-year period, 15 patients had a relapsing course (6.8%). Their ages ranged from 8–70 years. They had 36 episodes at a variable interval of 3 months to 25 years. Relapse rate varied from 1–4. Individual episodes were similar to the acute monophasic illness, although they differed in severity from one episode to the other[107].

## The Origin of "Noninfectious" Mosaics

A mosaic of manifestations is a general characteristic of every kind of noninfectious disease. Identifying the origin is great, since it may provide novel targets for preventative and therapeutic measures that are desperately needed.

Although only cumulated evidence suggests that inherited genetic variation plays a role in the pathogenesis of noninfectious diseases, heritability is the less-disputable paradigm about its origin. Nevertheless, although the identification of susceptibility genes for this kind of disease is a vital goal, the real progress in unearthing novel genes has been slow. For instance, the optimistic predictions of the impact of genetics on the diagnosis and prevention of atherosclerosis[108] generated enormous enthusiasm and expectations. Unfortunately, they could not been realized[109]. In reality, it is risky to imagine the existence of separate genes responsible for any individual localization, size, or intensity of development of atherosclerotic affection.

Classical genetics cannot explain why some atherosclerotic persons reveal only solitary asymptomatic plaques on the wall of a peripheral artery, whereas others suffer multiple plaques located in many arteries that provide very important organs with blood. In other words, which genetic factors are able to determine the small parts of the arterial wall that are receptive to atherogenic influence, while not injuring far more extensive neighboring parts of the same vessels? Thus, it becomes obvious that humans, i.e., different parts of their individual aortal circulatory systems, can reveal extraordinary genetic diversity in their predisposition to atherosclerosis.

According to the data from many experiments and observations, the unequal quality of analogous structures of the same genetic origin in an entire organism arises as a result of intraspecies hybridization[1,3,19,33,40,110], thus forming heterozygotic offspring. It is such genetic examples that challenged Mendel's first law. That is codominance, a kind of relationship between two dominant alleles of a gene pair in a heterozygote at which the alleles do not express a typical dominance/recessive relationship, but are both fully expressed.

The most evidence for the genetic origin of obesity comes from classic genetic analyses of pedigree, twin, adoption, and familial aggregation studies, which estimated an obesity heritability of 0.7–0.8. Obesity tends to run in families, suggesting a genetic cause of the disease. Racial disparity in predisposition to obesity[111], interpopulation diversity in obesity[112], and inter- and intraindividual differences in manifestations of the disease confirm genetic predilection to obesity and open the possibility to decipher the origin of this kind of human individuality.

The genesis of these intraindividual diversities in the case of obesity can also be explained by hybridization of persons with different genetic immunity/susceptibility to internal factors determining obesity[49]. The mating of resistant and susceptible individuals gives rise to progeny with intermediate degrees of obesity and extent of obese foci. One can suppose that focal diversity in the rate and kind of dispersion of obesity manifestations is such as a result of hybridization between two genetically different organisms: one of them was constitutionally immune to leptin and insulin, whereas its mating partner was normally sensitive to these molecular physiological agents[49]. This conclusion is supported by direct observations that subjects who are heterozygous for the leptin mutation develop obesity that is not as severe as in homozygotes[113], and that variation in obesity-related traits is associated with interethnic genetic admixture[114].

The modern increase of obesity incidence in some countries can be associated with increased hybridization between representatives of populations with different genetic makeup that, in turn, could provide functional incomplementariness between a hormone and its receptor, thus determining the state of leptin deficiency. At least four variants of such incompatibility could be considered here[49]:

1. Aboriginal hormone meets mutant aboriginal receptor
2. Mutant aboriginal hormone meets aboriginal receptor
3. "Alien" hormone meets aboriginal receptor
4. Aboriginal hormone meets "alien" receptor

These data provide a starting point for the clinical, molecular, and genetic analysis of the obesity cases affecting different populations.

The existing set of observations allows us to conclude that the spread of obesity is determined by a rise of heterozygosity in the population under observation. Extraordinary diversity in the obesity course, the manifestations and severity of specific affections, and their sizes and focal disposition around the body could be created by interethnic hybridization (xenogamy) of persons with different grades of hormone-receptor heterozygosity. Prerequisites for the intensive performance of this mechanism arise everywhere in the world today, especially among more mixed populations. Heterozygous individuals of these populations who inherit variants that predispose them to disease-related traits have a greater chance of acquiring the disease of obesity[112,114].

This analogy allows us to suppose the same genetic origin in the case of intraindividual diversity in senescence, which can be explained by hybridization of persons with different genetic susceptibility to internal or/and environmental factors determining aging. It is possible that another explanation can be proposed, but for us, the above interpretation is more acceptable today.

The increase of the population and intraindividual heterogeneity as a result of migration processes is one of the factors contributing to greater variation of schizophrenia forms[115]. In addition, structural investigations have suggested that schizophrenia is associated with various cerebrocraniofacial dysmorphogenesis including cerebral asymmetry[116] usually induced by heterozygosity. Such a leading symptom of schizophrenia like mental splitting, as well as a positive correlation between the "complexity" of a society (mosaic heterozygosity of human populations) and the number of schizophrenics it allows, witnesses the vital necessity for specialized investigations in this direction[86].

Future findings on the pathogenesis of schizophrenia can be induced by disclosure of functional cytoecological dysfunction, dependent on constitutional incongruence between molecular neurotransmitters and their receptors possessed by outwardly homogeneous clones of different molecular phenotypes, distinguished by tiny, but very important, features of their molecular constitution. That incongruence is supposed to arise as a result of mating between people whose previous molecular evolution had performed differently in ecologically different environments, resulting in different quantities of relevant cell receptors, for instance[49].

All autoimmune affections also present focal syndromes, even in a case of their systemic distribution. They often affect many organs throughout the body including the skin, brain, spinal cord, nerves, blood vessels, heart, lungs, and muscles. Autoimmune aggression can affect almost any part of the body, i.e., any tissue of any organ or body system. Sometimes, it can affect many regions of a body, but it can never affect all parts of a tissue at once. Extraordinary diversity in the locations and sizes of the affections is characteristic of every kind of autoimmune disease. The origin and development of “nonself” parts inside of a body during its postnatal ontogenesis forms the main question of autoimmunity pathogenesis.

The exact mechanisms that cause autoimmune disorders are not clear, and the past decades of research on the lymphatic immune system have not seen substantial progress in our understanding of autoimmunity. Autoimmune diseases are considered as multigenic. Meanwhile, the evidence for genetic involvement in the pathogenesis of autoimmune diseases comes only from the studies of twins; really, in the case of monozygotic twins, both twins are more frequently affected than in the case of dizygotic twins. It is also accepted that autoimmune diseases arise as a result of aberrant misrecognition of some “self”-epitopes of a body as “nonself” by the lymphatic immunogenic system, which begins to mistakenly attack some parts of the body's own tissue and kills them like germs inducing inflammation and tissue damage.

It is also proposed that in the case of autoimmune aggression, the lymphatic immunogenic system in afflicted individuals selectively loses the ability to distinguish “self” from “nonself” and switchs "modes", from protective to attacking. Paradoxically, the inability to distinguish “self” from “nonself” is not manifested in all parts of an afflicted individual, but only in some of them. Some parts of the same cellular category are considered as “self” by the lymphatic system, while other parts of the same kind are considered “nonself”. The existent hypothesis of autoimmune aggression cannot explain the spotty distribution of relevant lesions.

Contrarily, one can suppose that some focally dispersed parts of an afflicted organism become antigenically “nonself’’ at the latest postnatal ontogenesis, whereas the lymphatic system continues to perform its innate functions that have been formed at prenatal ontogenesis. Autoimmune diseases arise as a result of the attack of the lymphatic system against aberrant epitopes emerged in a body during the latest postnatal ontogenesis.

Some subpopulations of cells that are formed in the course of postnatal ontogenesis appear to be “nonself” for the lymphatic system of immunogenesis, thus provoking the immune self-aggression. In a case of hormone adaptive systems that are of normal function, the self-aggression is restrained. On the contrary, in consequence of stress, age transformation, and other immune depressive factors, the defensive potential of hormone adaptive systems diminishes, allowing immune self-aggression.

There is a popular fallacy that all an individual’s somatic cells are genetically analogous and so they do not have conflicting interests. In reality, they may have different phenotypes and, therefore, different interests. Such a symbiotic coexistence of genomes forms the main source of the diversity, both in infectious and noninfectious diseases. The basis for diversity in both kinds of diseases is formed by the organism’s heterozygosity, resulting in the coexistence in the body of two active allelomorphic genes and of two heterogeneous parts of the body, for instance, of its organs, tissues, or cell clones. Molecular composition of such heterozygotes in some parts of a body is formed by the genotype of one parent, whereas in neighboring parts of the same body, its formation is controlled by the genotype of another parent. One of these heterogenous parts possesses the feature of one parent (e.g., genetic immunity to a disease’s inducing agent) whereas the other one possesses the alternative feature of other parent (i.e., genetic susceptibility to this agent).

Because the heterozygotes have their own mosaic phenotypes, this gives rise to different proportions among their offspring. Individual grades of heterozygosity may be extraordinarily variable. In the simplest cases of hybridization, the quotes of between-spouse admixture can consist either of 50:50%, 75:25%, 87.5:12.5%, or 93.75:6.25% of the whole parent’s genome. In addition, each of these quotes may be stochastically dispersed around the body, making extraordinarily different forms of the admixture.

The individual diversity in both infectious and noninfectious diseases is predestined by the organism’s heterozygosity, resulting in coexistence in the body of two active allelomorphic genes and of two allelic cell clones. Both of these alleles function dominantly. The heterozygous individual shows both alleles expressed equally, although in different locations of the body. The diversity in the danger and severity of diseases depends exclusively on the differences in the genetically predetermined variations in the quantity of susceptible cells in the body under consideration.

## The Functions of Intraindividual Mosaics

Intrapopulation differences in susceptibility to a pathogenic agent determine the variations in the spread of its epidemics. Like an immune individual, the immune population does not allow the agent to enter into it and spread around it. Immunity of a population, especially one of genetic origin, forms the mightiest barrier to the spread of relevant epidemics around this population. In contrast, the existence in a population of a relevant number of partially susceptible persons predisposes the population to rapid spreading of relevant epidemics. The existence of genetic immunity in a population performs the mightiest antiepidemic defense, whereas the biodiversity in the susceptibility to a disease functions as a savior of epidemic processes.

The intensity of any epidemic process, i.e., its ebb or flow, is predestined by the number of susceptible members in the relevant species as well as by the grades of their susceptibility.

The diversity in different kinds of noninfectious diseases can be of a different genesis. First, some diseases that are considered to be “noninfectious”, e.g., atherosclerosis, do not show principal pathogenetic differences from infectious ones. But in contrast to many infectious diseases, the diversity in grades of danger and severity of atherosclerosis depends not on the quantity of specific affections, but on its location.

Second, senescence (aging) should be considered as a result of the normal physiological performance of the genetically programmed process of individual ontogenesis. The diversity in senescence begins from species, populations, and individual and intraindividual differences in the level of the age where and when this process has been programmed to be finished. Besides, nobody died of absolute senescence being free of some diseases and their consequences.

Third, the epidemiological and clinical diversity in metabolic diseases, such as obesity, diabetes, rickets, testicular feminization, Cushing's disease, the syndrome of cell immunity to thyroid hormone, is associated with genetic resistance to relevant molecular physiological agents, i.e., hormones or mediators. The principles of cell immunity to physiological agents are analogous to those in the constitutional (genetic) immunity to infections that is also formed by structural incongruence between the relevant agent and its cell receptors. One could suppose this immunity could arise thanks to mutation, but it is absolutely impossible to imagine that those pathogenic mutations can be distributed around populations by direct natural selection for immunity to relevant molecular physiological agents.

Because many hormones share their specific targets with the acting molecules of some infectious agents, the evolutionary formation of constitutional immunity against an infection forms immunity to relevant physiological agents. Thus, the susceptibility to metabolic noninfectious diseases is formed by insusceptibility to some infections. It arises and spreads in populations as a result of the spread of genetic immunity to a relevant infectious agent. This kind of intermolecular incongruence can be reinforced by interethnic admixture. Thus, the mutant formation of the life-saving genetic immunity to infection can create a life-threatening immunity to the relevant hormone. The individual diversity functions as a savior of populations, whereas the intraindividual diversity functions as a savior of individual life.

The analogical way of pathogenesis can be discussed also in relation to schizophrenia. It can be induced by cell immunity to neurotransmitters. This kind of pathology could arise and spread in a population as it has been hypothesized in relation to the obesity's pathogenesis[49]. Nevertheless, the direct postinfectious origin of the disease also cannot be excluded for a while. In this case, one can propose innate differences in cell immunity to an infection, resulting in the death of a susceptible clone. This kind of biodiversity presents the most general feature of any kind of pathology.

In analogy to infectious diseases, the individual diversity in the grades of danger and severity of metabolic diseases depends directly on the quantity of specific affections and its location. The diversity in epidemic and clinical manifestations of metabolic diseases depends on the existence of their not deadly, but chronic, forms that provide the preservation of this kind of noninfectious pathology.

The integrated view on different levels of diversity in both infectious and noninfectious diseases shows new direction toward the deciphering of many actual aspects of epidemiology, etiology, pathogenesis, prophylaxis, diagnostics, and treatments being unopened until now. Whereas the possibility to be affected by an infection depends on both infectious agents and the attacked organism, the severity of the disease and its outcome depend exclusively on the genetic makeup, namely, on the grade of the attacked organism diversity.

Human reproductive ecology is changing dramatically, and these changes have manifestations in the terms of diseases and their distribution. High rates of current outbreeding, migration, and cross-ethnic mating will reassort human genes at unprecedented rates. As a result, the rates of diversity and the number of noninfectious diseases will rise. Genetic admixture forms the main moving force for the worldwide epidemics of noninfectious diseases. The existing grade of infectious diversity ensures that the modern world population consists of enough variations among individuals to provide the world population with genetic resistance to any newly emerging or re-emerging infectious disease, as well as against biological weapons[23]. The growth of diversity will ensure this kind of biodefense.

## CONCLUSIONS

Every human body consists of coexisting, phenotypically dissimilar, mosaic populations of homogenous cells of unequal molecular constitution. The phenotypic manifestations of the mosaicism are being formed during an individual's life, along with the realization of an individual program of ontogenesis. The investigation performed above presented an initial perspective of a special kind of diversity, which is characteristic of both infectious and noninfectious pathology.

In the light of data considered and generalized above, it became clear that manifestations of this multilevel kind of biodiversity may be observed beginning from species to populations, individuals, organs, tissues, cells, and finally to their organelles and molecular structures. In addition, one should strongly accentuate, that the beginning of this biodiversity is rooted on a molecular level. Any disease, either infectious or noninfectious, arises as a result of specific intermolecular interactions.

Both infectious and noninfectious diseases are of genetic predisposition, strongly connected with molecular features of constitutional immunity that are exceptionally specific for each disease. At the same time, the functions of this immunity in the discussed kinds of diseases are diametrically opposite. In the case of any infectious disease, constitutional immunity provides the attacked body with life-saving resistance, while in the case of undoubtedly noninfectious diseases (obesity, diabetes, other hormonal dysfunctions, autoimmune aggression, cancerogenesis, and probably the diseases of the mind), constitutional immunity forms principal prerequisites for their development. Atherosclerosis, senescence, and some other diseases of undetermined etiology may be considered as exceptions of these gradations.

The clinical diversity in both infectious and noninfectious diseases arises as a result of mixed sexual self-reproduction that forms these grades of heterozygosity. Any case of this diversity that has been considered above is such, as a result of hybridization between two genetically different organisms: one of them was constitutionally immune to relevant ecological or physiological agent, whereas its mating partner was constitutionally sensitive to it. As a result, the descendant's body cell clones are formed under control of two codominant allelomorphic genes. The first step of this hybridization is revealed as the dissimilarity of molecular constitution of homologous cells and their subcellular structures.

The phenomenon of heterozygosity cannot be considered as something unusual. Heterozygosity and relevant intraindividual diversity are intrinsic of sexual reproduction. Even in a case of inbreeding, both male and female genomes are not identical[117]. Males and females differ in their parental investment. Each sexual cooperator has had a different evolutionary history, and has both a different genotypic and phenotypic makeup. That is why each descendant of any couple is heterozygotic even in the case of inbreeding, and thus can be considered at least as a bigenome organism. The kind of intraindividual diversity can manifest itself either on molecular and cellular levels, or on the levels of tissues and organs. This may lead to the differences and, sometimes, to conflicts inside the descendant's phenome.

In a case of outbreeding, the grades of heterozygosity are especially variable. Each heterozygotic body is formed and functions under control of two genomes mixed in individually different proportions. As a result of heterozygosity, within a multicellular organism, phenotypically dissimilar clones of homogeneous cells and extracellular structures with unequal molecular constitutions coexist. The clones vary in their liability to relevant exogenous and endogenous factors. They exist in a body in the form of separated populations dispersed either stochastically or legitimately around the body in mosaic patches.

The correlation between those dissimilar clones within the specimens of one species is very changeable, and it is the reason for the organism's varied individual predisposition to different diseased states in respect of their spread, relevance, course, and severity. Human populations are unprecedented in the number of differences of their genetic and phenotypic makeup. The genome of a modern civilized human can be considered as a mosaic composed of genetic segments inherited from a long row of different ancestors.

In these circumstances, thinking about the prophylaxis of obesity, diabetes, schizophrenia, and other diseases of "noninfectious" etiology, we should pause to remember the commandment of Abraham: "… you will not take a wife…from the daughters of the Ca'naan'ites in among whom " we are dwelling[118]. A question arises immediately about the tests for structural and functional congruence of genomes that should be elaborated and used today to prevent the formation of intraindividual incongruence in the offspring's body.

The anti-infectious prevention programs can be made far more effective by targeting them towards susceptible individuals. In agriculture, infectious diversity is a key tool in breeding new crop, fowl, and cattle varieties. A recent revival of research on genetic immunity has been stimulated by the recognition that both farming and human health care must become more effective. In contrast, the programs of prevention of noninfectious diseases should be targeted toward the restriction of relevant diversity.

The main applied results of discovery of the diversity in diseases disperse far out of the interests of medicine, agriculture, and biotechnology. Infectious diseases and their diversity are becoming increasingly recognized as having a major influence on the evolution of biological populations, species, and all life as a whole[18]. Although the systematic application of medicine to evolutionary biology is a very new and unaccustomed enterprise, medicine begins to inform biology of previously unknown principles of living beings' constitution and evolution.
